# The efficacy of acupuncture for post-stroke dysphagia: an overview of systematic reviews and meta-analyses

**DOI:** 10.3389/fnins.2025.1640471

**Published:** 2025-08-28

**Authors:** Fangyuan Xu, Xuepeng Wang, Fan Dai, Yu Ye, Xingxing Su, Peijia Hu, Hongliang Cheng

**Affiliations:** ^1^The First Clinical Medical School, Anhui University of Chinese Medicine, Hefei, China; ^2^Department of Neurosurgery, The First Affiliated Hospital of Anhui University of Chinese Medicine, Hefei, China; ^3^The Second Affiliated Hospital of Anhui University of Chinese Medicine, Hefei, China

**Keywords:** acupuncture, stroke, dysphagia, overview, meta-analyses

## Abstract

**Background:**

Acupuncture has been widely used in clinical rehabilitation as an adjunctive therapy for post-stroke dysphagia (PSD). Although numerous meta-analyses (MAs) have evaluated its efficacy, a comprehensive assessment of the methodological quality and evidence strength of these MAs is still lacking.

**Methods:**

Two researchers independently searched eight databases for relevant literature, screened studies according to predefined inclusion and exclusion criteria, and extracted data from the eligible systematic reviews (SRs) and MAs. The methodological quality, reporting completeness, risk of bias, and strength of evidence were rigorously evaluated using the AMSTAR 2, PRISMA-A, ROBIS, and GRADE, respectively. In addition, the GROOVE tool was used to assess the degree of overlap among original studies by calculating corrected covered area (CCA).

**Results:**

This overview included 19 MAs. Based on AMSTAR 2, four studies were rated as low quality, while 14 were rated as critically low quality. In terms of reporting quality, major deficiencies were observed, including a lack of protocol registration, incomplete search strategies, inadequate risk of bias assessments, and missing funding disclosures. For risk of bias, only six studies were judged to be at low risk. Furthermore, it revealed a slight overlap among the original studies with a CCA of 2.86%. Among the 68 outcome indicators, only 11.76% were graded as moderate quality, while 50% were classified as low quality and 38.24% as critically low quality, according to the GRADE assessment. Among the moderate-quality outcomes, electroacupuncture combined with swallowing rehabilitation therapy (SRT) demonstrated superior effectiveness compared to SRT alone (OR = 5.40, 95% CI: 3.78–7.72), as did acupuncture plus SRT (RR = 1.26, 95% CI: 1.19–1.34). Significant improvements in swallowing function were also reported, as measured by scales such as the Water Swallowing Test (WMD = −0.69, 95% CI: −0.78 to −0.60) and the Penetration Aspiration Scale (MD = −1.02, 95% CI: −1.27 to −0.78).

**Conclusion:**

While acupuncture appears to be a promising adjunctive treatment for PSD, the overall quality of evidence remains low. More rigorously designed and transparently reported studies are needed to strengthen the evidence base and support clinical decision-making.

## 1 Introduction

Swallowing is a complex physiological process that plays a critical role in human quality of life. It involves the coordinated and dynamic interaction of the oropharyngeal and esophageal muscles to ensure the safe and efficient transport of liquids and solids from the oral cavity to the stomach (Jones et al., 2020). Dysphagia is one of the most common complications following stroke, typically presenting as impaired swallowing function, difficulty in bolus transit, and coughing or choking when drinking. These symptoms can have significant adverse effects on both physical health and psychological well-being (Zhang B. et al., 2025). Although some patients with post-stroke dysphagia (PSD) may recover spontaneously within the first few weeks, a substantial proportion experience persistent swallowing difficulties for up to 6 months following the event (Sasegbon et al., 2025). Dysphagia significantly increases the risk of malnutrition and aspiration pneumonia, both of which are associated with elevated mortality in PSD patients (Cohen et al., 2016). Moreover, prolonged rehabilitation poses a considerable economic burden on individuals, families, and healthcare systems. Studies have indicated that only 17.6% of patients return to their pre-stroke dietary status following standard care (Carnaby et al., 2020). Therefore, there is an urgent need to develop and implement effective swallowing rehabilitation strategies to restore swallowing function, enhance daily living capabilities, reduce the incidence of aspiration, and ultimately optimize clinical outcomes for stroke survivors.

Conventional interventions for PSD primarily encompass behavioral therapies (e.g., the Mendelsohn maneuver), dietary modifications (such as adjustments to liquid viscosity and food texture), nutritional support (e.g., nasogastric tube feeding when oral intake is insufficient), and oral motor or sensory stimulation (Dziewas et al., 2021; Gómez-García et al., 2023). However, these therapeutic approaches often require varying degrees of patient cooperation, which may limit their applicability in individuals with impaired consciousness. In recent years, neurostimulation techniques, including pharyngeal electrical stimulation (PES), repetitive transcranial magnetic stimulation (rTMS), and transcranial direct current stimulation (tDCS), have gained attention for their potential to modulate neural pathways and enhance synaptic plasticity. One study found that PES increased the proportion of stroke patients with tracheostomies deemed ready for decannulation (Dziewas et al., 2018). Nevertheless, the generalizability of these findings remains uncertain, and there is currently insufficient evidence supporting the efficacy of PES in non-ventilated PSD patients (Dawson et al., 2024). Both rTMS and tDCS target the cerebral cortex to modulate cortical excitability, but therapeutic outcomes vary considerably depending on stroke location and the specific brain areas stimulated. Currently, there is a lack of robust evidence to determine the optimal stimulation targets and parameters for different lesion sites, which may limit the consistency and reliability of neurostimulation-based treatments.

As a key component of traditional medicine, acupuncture has gained increasing recognition for its potential role in stroke rehabilitation. In 2021, the European Stroke Organisation and the European Society for Swallowing Disorders issued a guideline moderately recommending acupuncture as a potentially beneficial intervention for improving swallowing function in patients with PSD (Dziewas et al., 2021). Acupuncture is believed to stimulate neuromuscular tissues, enhance local blood circulation, promote contraction of swallowing-related muscles, and help restore the swallowing reflex arc (Zhang et al., 2017). Administered alone or in combination with other rehabilitation strategies, acupuncture has shown promising benefits in improving swallowing function, enhancing quality of life, alleviating psychological distress, and contributing to better clinical prognosis in patients with PSD.

Systematic reviews (SRs) are structured evaluations of existing literature that apply explicit and systematic methods to identify, select, and critically appraise relevant studies, thereby aiming to generate reliable conclusions (Siddaway et al., 2019). Meta-analyses (MAs), as quantitative extensions of SRs, utilize statistical techniques to synthesize data from multiple studies to address specific research questions (Arya et al., 2020). When combined, SRs and MAs are regarded as the highest level of evidence in evidence-based medicine and serve as a cornerstone of clinical decision-making. However, low-quality SRs and MAs may yield misleading or unreliable results, underscoring the necessity of critical quality appraisal. In recent years, numerous SRs and MAs have examined the efficacy of acupuncture for PSD. Nevertheless, they vary considerably in methodological rigor, reporting quality, and certainty of evidence. This heterogeneity introduces uncertainty for clinicians and researchers seeking reliable, evidence-based conclusions. In this context, an overview that systematically collects, synthesizes, and critically appraises existing SRs and MAs offers a higher-level summary of the evidence to inform clinical practice and future research (Zhang D. et al., 2025). Accordingly, this overview evaluates existing SRs and MAs on acupuncture for PSD in terms of methodological quality, risk of bias, reporting quality, and certainty of evidence, aiming to identify limitations in the current evidence base and provide guidance for clinical decision-making.

## 2 Methods

### 2.1 Study registration

The protocol of this overview was registered in the International Prospective Register of Systematic Reviews (PROSPERO) at https://www.crd.york.ac.uk/PROSPERO/ with registration number CRD420251042911.

### 2.2 Search strategy

Two independent researchers (XFY and DF) conducted a comprehensive search across the following eight databases: PubMed, Web of Science, Embase, The Cochrane Library, China National Knowledge Infrastructure (CNKI), WanFang Database, China Science and Technology Journal Database (VIP), and SinoMed. The search covered records from database inception to April 7, 2025. Additional manual searches of reference lists and protocol registries were performed to ensure comprehensiveness. The search strategy combined Medical Subject Headings (MeSH) and free-text terms, with full details provided in Supplementary Table S1.

### 2.3 Inclusion criteria

(1) Study design: SRs and MAs focusing on acupuncture for the treatment of PSD; (2) Participants: Patients diagnosed with PSD based on widely accepted diagnostic criteria, with no restrictions on age, sex, ethnicity, stroke subtype, lesion location, or dysphagia severity; (3) Interventions: The intervention group received acupuncture therapies, including scalp acupuncture, electroacupuncture, warm acupuncture, manual acupuncture, or acupuncture combined with rehabilitation training or conventional treatment. The control group received rehabilitation training, Western medicine, or conventional treatment. For studies where the intervention group received acupuncture in combination with other therapies, the control group must have received the same co-interventions, excluding acupuncture; and (4) Outcome Indicators: Primary outcomes included total effective rate, water swallowing test (WST), videofluoroscopic swallowing study (VFSS), Fujishima dysphagia scale (FDS), standard swallowing assessment (SSA), dysphagia outcome severity scale (DOSS), penetration-aspiration scale (PAS), and hyoid bone displacement. Secondary outcomes included the swallowing quality of life (SWAL-QOL) questionnaire, Barthel index (BI), modified Barthel index (MBI), activities of daily living (ADL), and safety-related outcomes.

### 2.4 Exclusion criteria

(1) Duplicate publications, network meta-analyses, narrative reviews, study protocols, dissertations, or conference abstracts; (2) Studies involving PSD patients with comorbidities; (3) Studies in which acupuncture was not the primary intervention or was also included in the control group; (4) Studies where acupuncture was not the sole variable distinguishing the intervention from the control groups; (5) Studies for which the full text was not available; and (6) Studies with incomplete or insufficiently reported data.

### 2.5 Study screening and data extraction

All records retrieved from the eight databases were imported into NoteExpress for screening. Duplicate studies were identified and removed using the software’s duplication-check function. Subsequently, two systematically trained researchers (WXP and XFY) independently screened the remaining records by examining titles, abstracts, and keywords to identify potentially eligible studies. Full texts of the selected articles were then reviewed in detail to determine whether the SRs and MAs met the predefined inclusion criteria. The two researchers independently extracted relevant data from the included SRs and MAs, including publication information, the number of primary randomized controlled trials (RCTs), total sample size, intervention and control strategies, assessment tools for methodological quality, outcome measures, and main conclusions. Any disagreements during the screening or data extraction process were resolved through discussion; if consensus could not be reached, a third researcher (CHL) was consulted to make the final decision.

### 2.6 Overlap calculation of the SRs and MAs

The original RCTs included in the meta-analyses may exhibit varying degrees of overlap, potentially influencing the interpretation of results. The graphical representation of overlap for the overviews (GROOVE) tool can visualize the degree of overlap of literature by calculating the evidence matrix and corrected covered area (CCA) (Pérez-Bracchiglione et al., 2022). The CCA is calculated using the formula CCA = (N−r)/(rc−r), where N denotes the total number of original studies included across all SAs and MAs with duplicates counted, c represents the number of SAs and MAs, and r indicates the number of original studies. A CCA of less than 5% was considered to indicate a slight degree of overlap. A CCA between 5 and 10% was defined as moderate overlap, 10 to 15% as high overlap, and greater than 15% as very high overlap. To compute the CCA, the authors, publication years, and included primary studies from each SR were extracted and entered into the GROOVE Excel spreadsheet, which automatically calculates the CCA and provides a visual summary of the overlap.

### 2.7 Methodological quality assessment

A Measurement Tool to Assess Systematic Reviews 2 (AMSTAR 2) will be applied to appraise the methodological quality of the included SRs. The tool comprises 16 items, seven of which are considered critical domains (items 2, 4, 7, 9, 11, 13, and 15). Each item will be evaluated as “Yes,” “Partially Yes,” or “No” based on the degree of adherence to methodological standards. Particular attention will be paid to the critical items, as they heavily influence the overall quality rating. Based on the evaluation, the overall methodological quality of each SR will be categorized as “High,” “Moderate,” “Low,” or “Critically low” (Shea et al., 2017). Two reviewers will independently perform the assessments and subsequently cross-check their results. Any divergence will be dealt with after a team discussion or an independent decision from a third reviewer.

### 2.8 Evaluation of reporting quality

The Preferred Reporting Items for Systematic Reviews and Meta-analyses of Acupuncture (PRISMA-A) checklist (Wang et al., 2019), introduced in 2019, serves as an extension for researchers to appraise the reporting quality of SRs about acupuncture. The checklist consists of 27 items covering the title, abstract, introduction, methods, results, discussion, and funding. Each item is evaluated as “yes” (fully reported), “partially yes” (partially reported), or “no” (not reported), and the overall compliance is expressed as a proportion.

### 2.9 Assessment of risk of bias

The Risk of Bias in Systematic Reviews (ROBIS) tool was used to assess the risk of bias in the included SRs. This process involves three phases: (1) assessing relevance (as appropriate); (2) determining the extent of bias risk during the systematic review process; and (3) making an overall judgment on the risk of bias in the review. Each phase includes several signaling questions, with responses categorized as “yes,” “probably yes,” “no,” “probably no,” or “no information.” Based on these responses, the overall risk of bias in each review is classified as “low,” “high,” or “unclear” (Whiting et al., 2016).

### 2.10 Evaluation of the quality of evidence

Two researchers (DF and YY) will independently evaluate the quality of evidence using the Grading of Recommendations Assessment, Development, and Evaluation (GRADE) approach. Evidence from RCTs is initially considered high quality but may be downgraded one or two levels for the condition of limitations of the methodological quality, inconsistency and imprecision of the results, indirectness of the evidence, and the potential publication bias (Brozek et al., 2009). For each outcome, the overall quality of evidence will be rated using GRADE profiler software and classified into four levels (high, moderate, low, or very low) based on the above downgrading factors. Discrepancies between the two reviewers will be resolved by another author (HPJ) to reach an agreement.

## 3 Results

### 3.1 Literature selection

**Figure 1:**
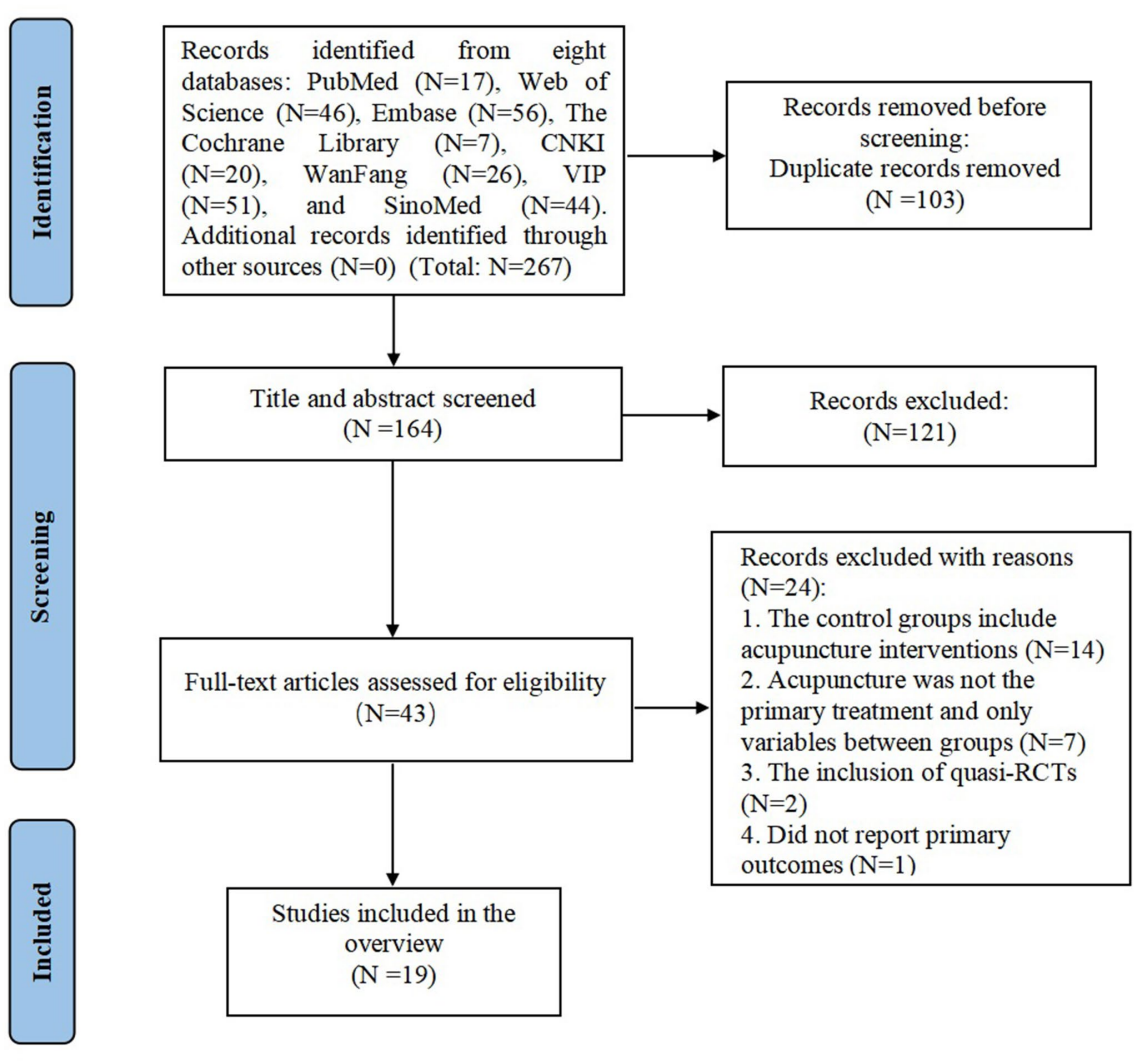
The flow diagram of the literature selection.

A total of 267 records were initially identified through comprehensive searches of eight Chinese and English databases. After removing 103 duplicates, 121 records were excluded based on title and abstract screening. The full texts of the remaining 43 articles were then assessed for eligibility. Among these, 14 studies were excluded because acupuncture was included in the control group, 2 because acupuncture was not the primary intervention, 5 because acupuncture was not the sole differing variable between intervention and control groups, 2 were quasi-randomized controlled trials, and 1 did not report primary outcome measures. Ultimately, 19 SRs and MAs were included in the final analysis. The study selection process is detailed in Figure 1.

### 3.2 Study characteristics

The 19 included SRs and MAs (Long and Wu, 2012; Yu et al., 2016; Wang et al., 2017; Ye et al., 2017; Li et al., 2018, 2021, 2024; Wu et al., 2018; Li and Deng, 2019; Zhao et al., 2019; Huang et al., 2020; Geng et al., 2021; Lu et al., 2021; Wang et al., 2021; Zhong et al., 2021; Jiang et al., 2022; Tang et al., 2022; Wang et al., 2022; Zhang J. et al., 2025) were published between 2013 and 2025, with 12 articles in English and 7 in Chinese. The number of included randomized controlled trials (RCTs) in these reviews ranged from 9 to 72, with sample sizes varying from 965 to 6,134 participants. The acupuncture interventions in the treatment groups encompassed various methods such as manual acupuncture, electroacupuncture, nape acupuncture, and acupressure. Control groups primarily received swallowing rehabilitation therapy, conventional treatment, or medication. Outcome measures were generally categorized into three domains: overall effectiveness, swallowing function, and quality of life or activities of daily living. Swallowing function was assessed using tools such as WST, VFSS, FDS, SSA, DOSS, PAS, and hyoid bone displacement. Adverse events were reported in eight of the included studies. Regarding methodological quality assessment, 14 SRs and MAs employed the Cochrane Risk of Bias tool, 4 used the Jadad scale, and only 1 referred to the CONSORT and STRICTA checklists. Key characteristics of the included 19 SRs and MAs, including sample sizes, intervention types, main outcome indicators, quality assessment tools, and conclusions are summarized in Table 1.

**Table 1:** Characteristics of the included SAs and MAs.

Author (year)	Country	Number of RCT (sample size)	Treatment intervention	Control intervention	Main outcomes	Quality assessment tool	Overall conclusion
Geng et al. (2021)	China	18 (1352)	EA + SRT	SRT	①②③④⑤	Cochrane	EA can improve swallowing function and has a good therapeutic effect on PSD, with good safety.
Huang et al. (2020)	China	16 (1216)	EA + SRT	SRT	①②③④⑤⑥	Cochrane	EA combined with SRT treatment for PSD patients can help improve clinical efficacy.
Jiang et al. (2022)	China	33 (2680)	Acupuncture (+SRT)	SRT	②③④⑤⑥⑦⑧⑨⑩⑪⑫⑬	Cochrane	It provided positive evidence that acupuncture or acupuncture combined with rehabilitation approaches for PSD is superior to using rehabilitation treatment alone.
Li et al. (2018)	China	29 (2190)	Acupuncture (+SRT)	SRT	①	CONSORT and STRICTA checklist	This study recommends acupuncture and moxibustion as an effective and safe alternative treatment for dysphagia after stroke.
Li and Deng (2019)	China	17 (1479)	Acupuncture+SRT	SRT	①②③④⑧⑨⑩⑪	Cochrane	Acupuncture combined with SRT may improve the effective rate, swallowing function, and activities of daily life of patients with PSD compared with SRT alone.
Li et al. (2021)	China	30 (2446)	Acupuncture/EA + SRT(+CT/Cold stimulation/electrical stimulation)	SRT (+CT/Cold stimulation/electrical stimulation)	①②⑥⑧	Cochrane	Acupuncture has better therapeutic effects on PSD compared to other treatment methods.
Li et al. (2024)	China	16 (1284)	Acupuncture/EA (+SRT)	SRT	①③⑤⑭⑮	Cochrane	Acupuncture combination therapy and acupuncture alone can effectively improve aspiration caused by PSD, with a low incidence of adverse events.
Long and Wu (2012)	China	72 (6134)	Acupuncture+SRT/CT	SRT/CT	①	Jadad	Acupuncture may help the rehabilitation of stroke patients affected by dysphagia.
Lu et al. (2021)	China	39 (3207)	Acupuncture+SRT	SRT	①②③④⑧⑪	Cochrane	The existing evidence supports that acupuncture therapy can significantly improve the swallowing function of patients with PSD.
Tang et al. (2022)	China	11 (1069)	Nape acupuncture+SRT	SRT	①③⑤⑧	Jadad	Nape acupuncture combined with SRT is more effective in treating PSD than rehabilitation alone.
Wang et al. (2017)	China	32 (2831)	Acupuncture/EA/ Nape acupuncture+SRT/medication	SRT/medication	①	Jadad	Acupuncture treatment for PSD has good clinical efficacy.
Wang et al. (2021)	China	14 (954)	Acupuncture/EA/ Nape acupuncture+SRT (+CT)	SRT (+CT)	①②⑧	Cochrane	Acupuncture combined with swallowing training can promote the recovery of swallowing function in PSD patients.
Wang et al. (2022)	China	7 (637)	Acupuncture (+SRT)	SRT	①	Cochrane	Acupuncture can effectively treat swallowing difficulties caused by pseudomedullary paralysis after stroke. The effect of acupuncture combined with SRT performs better.
Wu et al. (2018)	China	14 (1070)	Acupressure+SRT	SRT	①②④⑥	Cochrane	Acupressure is beneficial for improving swallowing disorders after stroke.
Ye et al. (2017)	China	71 (6010)	Acupuncture+SRT + CT	SRT + CT	①②③④⑤⑧⑩	Cochrane	The acupuncture group showed better efficacy in reducing swallowing dysfunction after stroke compared to the control group.
Yu et al. (2016)	China	9 (965)	Acupuncture+SRT	SRT	①②④	Jadad	Acupuncture has a satisfactory therapeutic effect on PSD.
Zhang J. et al. (2025)	China	20 (1718)	Acupuncture+SRT	SRT	②③⑤⑧⑪	Cochrane	The trial sequential analysis demonstrated the positive effects of acupuncture on swallowing function in PSD patients.
Zhao et al. (2019)	China	28 (2557)	Acupuncture+SRT + CT	SRT + CT	①⑥⑩⑪	Cochrane	Acupuncture treatment for PSD can improve the clinical efficacy of modern rehabilitation therapy and help improve patients’ daily living activities and quality of life.
Zhong et al. (2021)	China	35 (3024)	Acupuncture+SRT	SRT	②③④⑤⑧	Cochrane	Acupuncture may be an effective treatment for dysphagia after stroke.

EA, electroacupuncture; SRT, swallowing rehabilitation therapy; CT, conventional therapy; ① Clinical effective rate; ② Water Swallow Test (WST); ③ Videofluoroscopic Swallowing Study (VFSS); ④ Fujishima Dysphagia Scale (FDS); ⑤ Adverse events; ⑥ Incidence of aspiration pneumonia or pulmonary infection; ⑦ The rates of aspiration; ⑧ Standard swallowing assessment (SSA) scores; ⑨ The dysphagia outcome severity score (DOSS); ⑩ Activities of Daily Living (ADL), Barthel index (BI), modified BI; ⑪ Swallowing quality of life questionnaire (SWAL-QOL); ⑫ Duration of empty swallowing; ⑬ Duration of empty swallowing; ⑭ Penetration aspiration scale (PAS); ⑮ Hyoid bone displacement.

### 3.3 Overlap of primary studies

**Figure 2:**
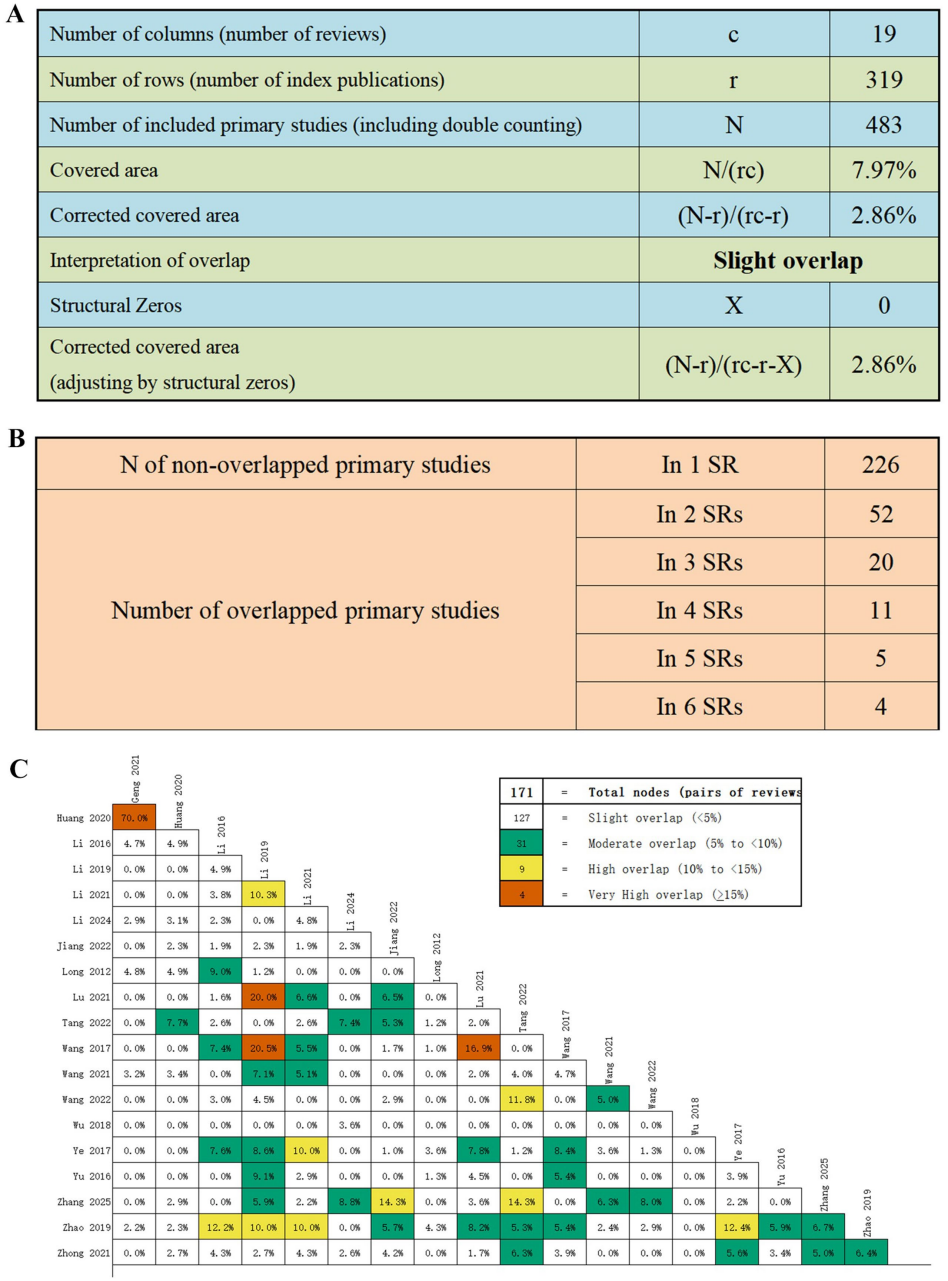
**(A)** Overall overlap results using the GROOVE tool; **(B)** Number of non-overlapped and overlapped primary studies; **(C)** Graphical representation of overlap for overviews.

Using the GROOVE tool, we quantified the degree of overlap among the primary studies included in the 19 SRs and MAs. The overall CCA was 2.86%, indicating only a slight overlap (Figure 2A). Across the 19 SRs and MAs, a total of 226 non-overlapping original studies were identified. Additionally, 52 duplicate studies were included in two SRs, 20 studies appeared in three SRs, while 11, 5, and 4 studies were duplicated across four, five, and six SRs, respectively (Figure 2B). Each node (box) in Figure 2C shows the overlap rate between two SRs. Among the 171 nodes, 127 demonstrated slight overlap, 31 moderate overlap, 9 high overlap, and only 4 exhibited very high overlap.

### 3.4 Methodological quality of the included SRs and MAs

**Figure 3:**
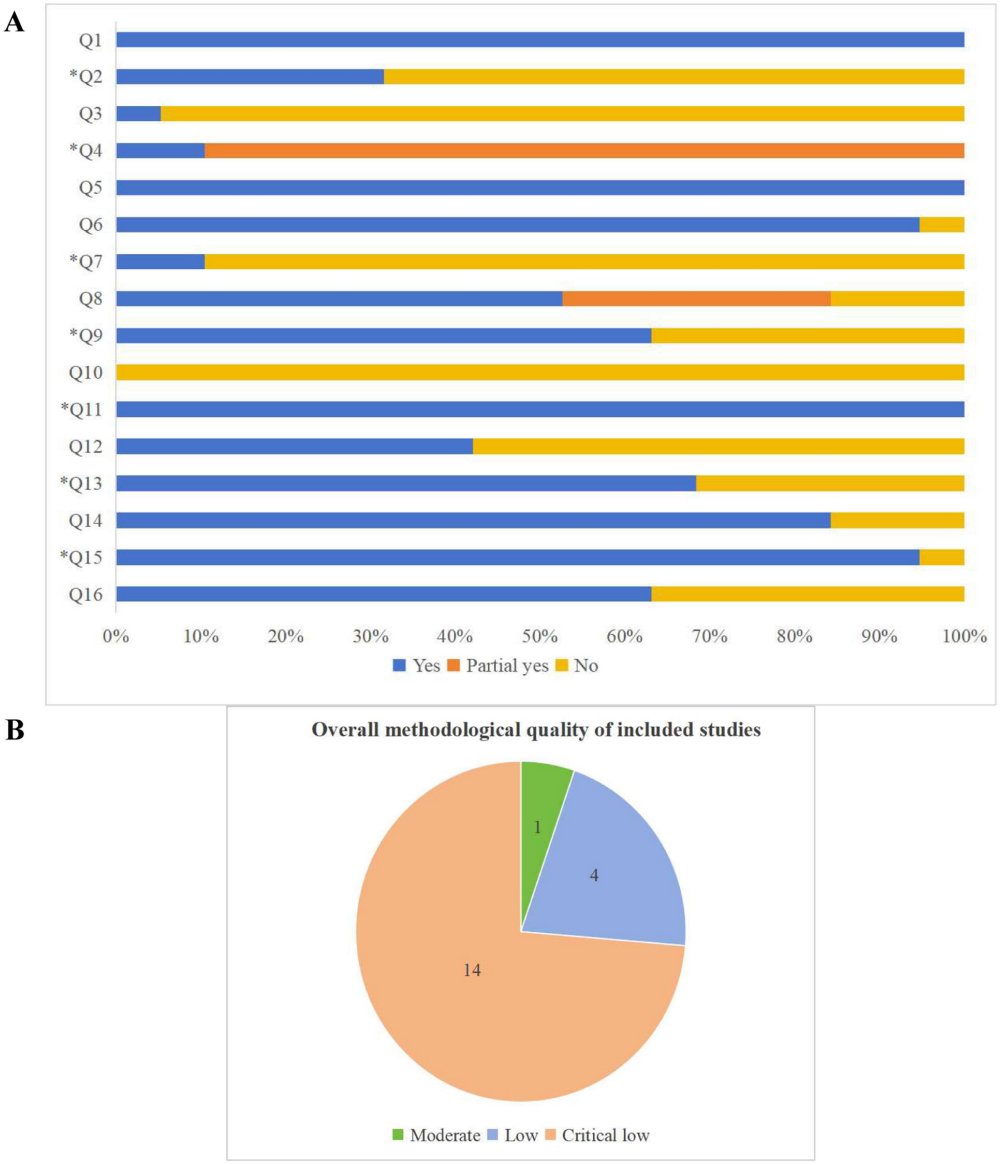
**(A)** The bar graph of the AMSTAR 2 assessment; **(B)** The pie chart of the overall methodological quality of the included SRs and MAs based on AMSTAR 2.

Methodological quality assessment of the included SRs and MAs using the AMSTAR 2 tool revealed that only one study was rated as moderate quality, indicating adherence to all critical domains but noncompliance with more than one noncritical item. Four studies were rated as low quality, while 14 were rated as critically low quality due to noncompliance with at least one critical domain (Figure 3B). Figure 3A provides a visual summary of compliance with individual AMSTAR 2 items across the included studies. Regarding specific methodological criteria, only six SRs and MAs were prospectively registered or published a study protocol. Seventeen studies searched multiple databases, reported detailed search strategies, and stated search limitations; however, only two studies reported searching the reference lists of included articles or clinical trial registries. In terms of transparency in study selection, only two SRs and MAs listed excluded studies along with the reasons for exclusion. Concerning risk of bias assessment, seven SRs and MAs did not report evaluating the risk of bias in the included RCTs. All studies employed appropriate statistical methods for data synthesis. When interpreting and discussing the results, 13 studies considered the potential influence of studies with differing levels of bias. With respect to publication bias, 18 studies assessed its presence and discussed its potential impact on the findings. Further details of the AMSTAR 2 assessments are provided in Table 2.

**Table 2:** The methodological quality of the included literatures assessed by AMSTAR-2.

Author (year)	Q1	Q2*	Q3	Q4*	Q5	Q6	Q7*	Q8	Q9*	Q10	Q11*	Q12	Q13*	Q14	Q15*	Q16	Overall quality
Geng et al. (2021)	Y	N	N	PY	Y	Y	N	PY	Y	N	Y	N	Y	N	Y	N	CL
Huang et al. (2020)	Y	N	N	PY	Y	Y	N	Y	Y	N	Y	N	Y	Y	Y	Y	CL
Jiang et al. (2022)	Y	Y	N	PY	Y	Y	N	Y	Y	N	Y	N	Y	Y	Y	Y	L
Li et al. (2018)	Y	N	N	PY	Y	Y	N	N	N	N	Y	N	N	Y	Y	Y	CL
Li and Deng (2019)	Y	N	Y	Y	Y	Y	N	Y	N	N	Y	N	N	Y	Y	Y	CL
Li et al. (2021)	Y	N	N	PY	Y	Y	N	Y	Y	N	Y	N	Y	N	Y	N	CL
Li et al. (2024)	Y	Y	N	PY	Y	Y	N	Y	Y	N	Y	Y	Y	Y	Y	Y	L
Long and Wu (2012)	Y	N	N	PY	Y	Y	N	N	N	N	Y	N	N	N	N	Y	CL
Lu et al. (2021)	Y	N	N	PY	Y	Y	N	Y	Y	N	Y	Y	N	Y	Y	Y	CL
Tang et al. (2022)	Y	Y	N	PY	Y	Y	N	Y	Y	N	Y	Y	Y	Y	Y	Y	L
Wang et al. (2017)	Y	N	N	PY	Y	Y	N	N	N	N	Y	N	Y	Y	Y	N	CL
Wang et al. (2021)	Y	N	N	PY	Y	Y	N	PY	Y	N	Y	Y	Y	Y	Y	N	CL
Wang et al. (2022)	Y	Y	N	PY	Y	Y	Y	PY	N	N	Y	Y	N	Y	Y	Y	CL
Wu et al. (2018)	Y	N	N	PY	Y	Y	N	Y	N	N	Y	N	Y	Y	Y	N	CL
Ye et al. (2017)	Y	N	N	PY	Y	Y	N	PY	Y	N	Y	Y	Y	Y	Y	Y	CL
Yu et al. (2016)	Y	N	N	PY	Y	N	N	PY	N	N	Y	Y	N	Y	Y	N	CL
Zhang J. et al. (2025)	Y	Y	N	PY	Y	Y	Y	Y	Y	N	Y	Y	Y	Y	Y	Y	M
Zhao et al. (2019)	Y	N	N	PY	Y	Y	N	PY	Y	N	Y	N	Y	Y	Y	N	CL
Zhong et al. (2021)	Y	Y	N	Y	Y	Y	N	Y	Y	N	Y	N	Y	Y	Y	Y	L

*Represents key questions in the AMSTAR 2; Y, yes; N, no; PY, partial yes; M, moderate; L, low; CL, critical low.

### 3.5 Reporting quality of included SRs/MAs

**Figure 4:**
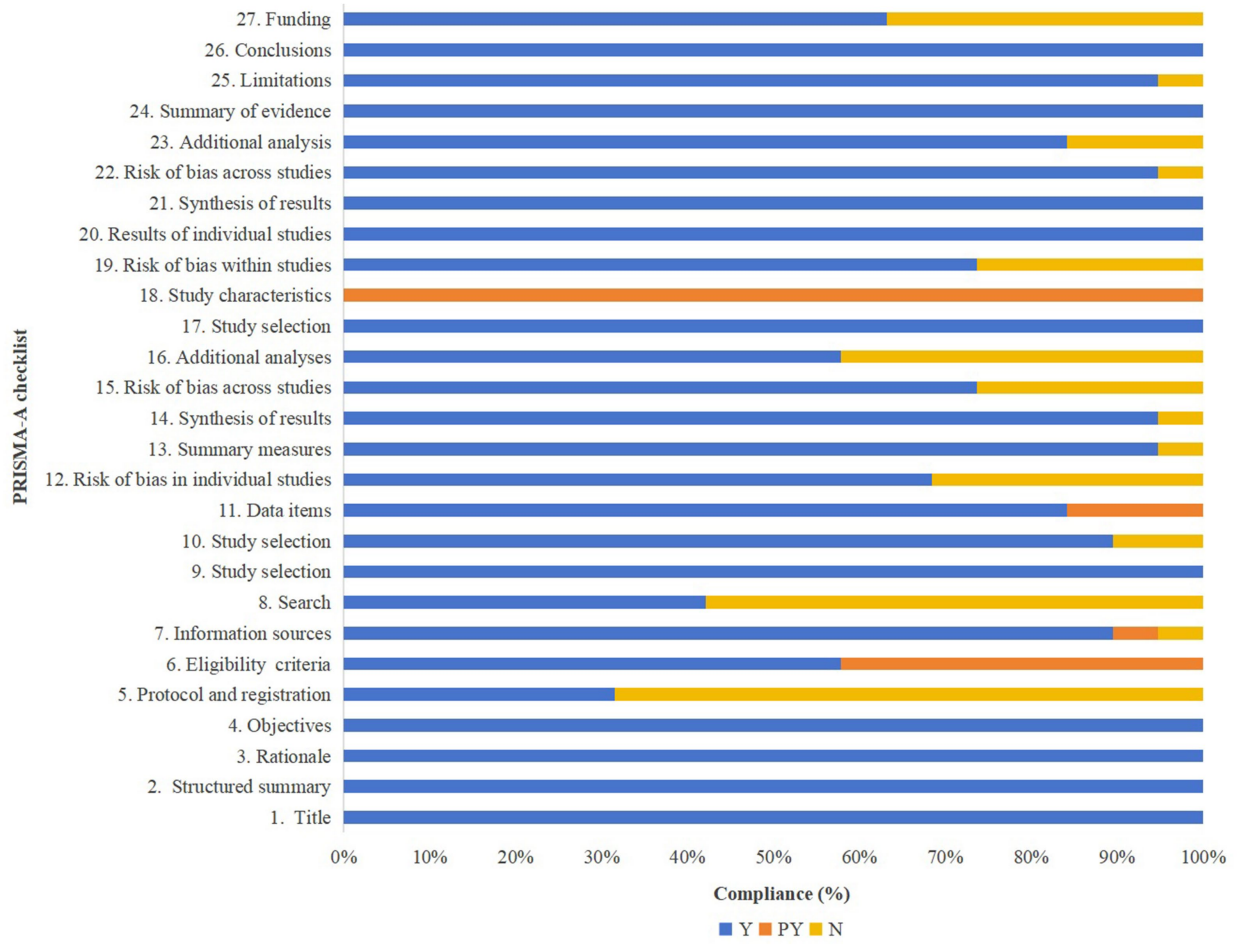
The bar graph of the PRISMA-A checklist.

The reporting quality of the included SRs and MAs, as assessed using the PRISMA-A checklist, is illustrated in Figure 4, with detailed evaluations provided in Table 3. All studies demonstrated full compliance with the items related to the title, abstract, and introduction sections. In the methods section, only 31.58% of the studies reported prior registration of their review protocols. For Item 6 (Eligibility criteria), eight studies were only partially compliant, primarily due to insufficient reporting of traditional Chinese medicine diagnostic criteria and the specific acupuncture modalities employed. Additionally, only 42.11% of the studies provided a complete search strategy, rather than simply listing search terms. Items 7 (Information sources), 9 (Study selection), 10 (Data collection process), 11 (Data items), 13 (Summary measures), and 14 (Synthesis of results) had completion rates exceeding 80%. Regarding risk of bias, 68.42% of the reviews described the methods used to assess the risk of bias in individual studies, while 73.68% evaluated the risk of bias in the overall body of evidence. Furthermore, 57.89% of the reviews conducted additional analyses such as sensitivity analyses, subgroup analyses, or meta-regression.

**Table 3:** The detailed information about the PRISMA-A checklist.

Sections	Items	Studies																			Percentage of yes and partially yes (*n*, %)
		1	2	3	4	5	6	7	8	9	10	11	12	13	14	15	16	17	18	19	
Title	Title	Y	Y	Y	Y	Y	Y	Y	Y	Y	Y	Y	Y	Y	Y	Y	Y	Y	Y	Y	19, 100%
Abstract	Structured summary	Y	Y	Y	Y	Y	Y	Y	Y	Y	Y	Y	Y	Y	Y	Y	Y	Y	Y	Y	19, 100%
Introduction	Rationale	Y	Y	Y	Y	Y	Y	Y	Y	Y	Y	Y	Y	Y	Y	Y	Y	Y	Y	Y	19, 100%
	Objectives	Y	Y	Y	Y	Y	Y	Y	Y	Y	Y	Y	Y	Y	Y	Y	Y	Y	Y	Y	19, 100%
Methods	Protocol and registration	N	N	Y	N	N	N	Y	N	N	Y	N	N	Y	N	N	N	Y	N	Y	6, 31.58%
	Eligibility criteria	PY	Y	Y	Y	PY	Y	Y	PY	PY	PY	PY	Y	PY	Y	Y	Y	Y	PY	Y	19, 100%
	Information sources	Y	Y	Y	Y	Y	Y	Y	Y	N	Y	Y	Y	Y	Y	Y	Y	Y	PY	Y	18, 94.74%
	Search	N	Y	Y	N	Y	N	Y	N	N	Y	N	Y	N	Y	N	N	Y	N	N	8, 42.11%
	Study selection	Y	Y	Y	Y	Y	Y	Y	Y	Y	Y	Y	Y	Y	Y	Y	Y	Y	Y	Y	19, 100%
	Data collection process	Y	Y	Y	Y	Y	Y	Y	Y	Y	Y	Y	Y	Y	Y	Y	N	Y	N	Y	17, 89.47%
	Data items	Y	Y	Y	Y	Y	Y	Y	Y	Y	Y	Y	Y	Y	Y	PY	PY	Y	PY	Y	19, 100%
	Risk of bias in individual studies	Y	Y	Y	N	N	Y	Y	N	Y	Y	N	Y	Y	Y	Y	N	Y	N	Y	13, 68.42%
	Summary measures	Y	Y	Y	Y	Y	Y	Y	Y	Y	Y	Y	Y	Y	Y	N	Y	Y	Y	Y	18, 94.74%
	Synthesis of results	Y	Y	Y	Y	Y	Y	Y	Y	Y	Y	Y	Y	Y	Y	N	Y	Y	Y	Y	18, 94.74%
	Risk of bias across studies	Y	Y	Y	Y	Y	Y	Y	N	Y	N	N	Y	Y	Y	N	Y	Y	Y	N	14, 73.68%
	Additional analyses	Y	N	Y	Y	Y	Y	Y	N	N	Y	N	N	Y	Y	N	N	Y	N	Y	11, 57.89%
Results	Study selection	Y	Y	Y	Y	Y	Y	Y	Y	Y	Y	Y	Y	Y	Y	Y	Y	Y	Y	Y	19, 100%
	Study characteristics	PY	PY	PY	PY	PY	PY	PY	PY	PY	PY	PY	PY	PY	PY	PY	PY	PY	PY	PY	19, 100%
	Risk of bias within studies	Y	Y	Y	N	N	Y	Y	N	Y	Y	N	Y	Y	Y	Y	N	Y	Y	Y	14, 73.68%
	Results of individual studies	Y	Y	Y	Y	Y	Y	Y	Y	Y	Y	Y	Y	Y	Y	Y	Y	Y	Y	Y	19, 100%
	Synthesis of results	Y	Y	Y	Y	Y	Y	Y	Y	Y	Y	Y	Y	Y	Y	Y	Y	Y	Y	Y	19, 100%
	Risk of bias across studies	Y	Y	Y	Y	Y	Y	Y	N	Y	Y	Y	Y	Y	Y	Y	Y	Y	Y	Y	18, 94.74%
	Additional analysis	N	Y	Y	Y	Y	N	Y	Y	Y	Y	Y	Y	Y	Y	Y	Y	Y	N	Y	16, 84.21%
Discussion	Summary of evidence	Y	Y	Y	Y	Y	Y	Y	Y	Y	Y	Y	Y	Y	Y	Y	Y	Y	Y	Y	19, 100%
	Limitations	Y	Y	Y	Y	Y	Y	Y	Y	Y	Y	N	Y	Y	Y	Y	Y	Y	Y	Y	18, 94.74%
	Conclusions	Y	Y	Y	Y	Y	Y	Y	Y	Y	Y	Y	Y	Y	Y	Y	Y	Y	Y	Y	19, 100%
Funding	Funding	Y	Y	Y	N	Y	Y	Y	N	Y	Y	N	N	N	N	Y	Y	Y	Y	N	12, 63.16%

Y, Yes; PY, partially yes; N, No.

Studies: 1. Geng et al. (2021); 2. Huang et al. (2020); 3. Jiang et al. (2022); 4. Li et al. (2018); 5. Li and Deng (2019); 6. Li et al. (2021); 7. Li et al. (2024); 8. Long and Wu (2012); 9. Lu et al. (2021); 10. Tang et al. (2022); 11. Wang et al. (2017); 12. Wang et al. (2021); 13. Wang et al. (2022); 14. Wu et al. (2018); 15. Ye et al. (2017); 16. Yu et al. (2016); 17. Zhang J. et al. (2025); 18. Zhao et al. (2019); 19. Zhong et al. (2021).

In terms of results reporting, all studies clearly described the selection process and reasons for inclusion or exclusion using flow diagrams and accompanying narratives. For Item 18 (Study characteristics), although all studies reported the characteristics of the included randomized controlled trials, none provided detailed information about the “Deqi” sensation following acupuncture treatment. Most SRs and MAs assessed the risk of bias of the included RCTs, synthesized results using forest plots, and performed risk of bias assessments as well as additional analyses (e.g., subgroup or sensitivity analyses). The discussion sections were generally well reported; only one study failed to adequately discuss the limitations of its findings and the review process. Regarding the funding item, 12 studies reported sources of funding or other forms of support for the SRs and MAs.

### 3.6 Risk of bias of included SRs and MAs

The ROBIS tool is designed not only to assess the risk of bias in the process and interpretation of SRs and MAs but also to evaluate the relevance of the review question to the practical issues faced by its intended users. Phase 1 focuses on evaluating the alignment between the target question and the question addressed by the SR using the PICOS framework. In this study, all included SRs and MAs were judged to be at low risk of bias in Phase 1.

Phase 2 aims to assess the risk of bias in the conduct of the SR, covering four key domains, each of which includes several signaling questions to help identify potential sources of bias. In Domain 1 (Study eligibility criteria), all studies were rated as low risk. In Domain 2 (Identification and selection of studies), only 6 out of 19 studies were rated as low risk. The primary reasons for elevated risk included incomplete search strategies and the omission of searches in clinical trial registries, reference lists, and manual searches. Regarding Domain 3 (Data collection and study appraisal), 14 SRs and MAs were rated as low risk, while the remaining 5 were considered high risk. The high risk was mainly attributed to the use of the Jadad scale, which does not assess allocation concealment. Studies that used only the Jadad scale without additional evaluation of allocation concealment were considered to have overlooked a significant source of bias. In Domain 4 (Synthesis and findings), 10 SRs and MAs were rated as low risk. For the remaining studies, the high risk stemmed primarily from the lack of publication bias analysis, uncertainty regarding adherence to the pre-specified protocol, unstable results, and the failure to clearly address the risk of bias in the original studies when presenting or discussing the findings.

Phase 3 involves an overall judgment of the risk of bias. Six studies were judged as low risk, as they appropriately interpreted the biases identified in Phase 2, reasonably considered the relevance between the included studies and the SR research questions, and provided an objective and comprehensive interpretation of the statistical findings. Details of each phase and domain assessed by the ROBIS tool are presented in Table 4.

**Table 4:** ROBIS results of included SRs and MAs.

Studies	Phase 1	Phase 2				Phase 3
		1. Study eligibility criteria	2. Identification and selection of studies	3. Data collection and study appraisal	4. Synthesis and findings	Risk of bias in the review
Geng et al. (2021)	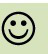	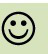	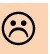	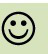	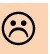	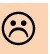
Huang et al. (2020)	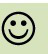	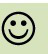	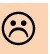	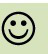	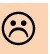	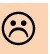
Jiang et al. (2022)	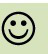	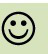	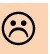	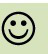	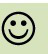	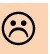
Li et al. (2018)	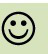	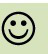	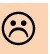	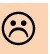	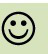	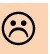
Li and Deng (2019)	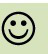	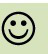	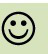	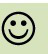	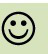	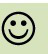
Li et al. (2021)	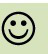	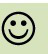	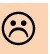	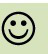	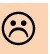	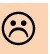
Li et al. (2024)	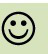	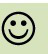	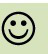	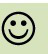	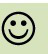	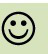
Long and Wu (2012)	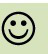	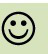	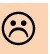	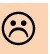	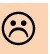	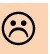
Lu et al. (2021)	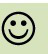	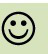	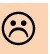	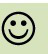	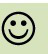	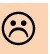
Tang et al. (2022)	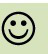	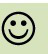	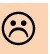	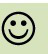	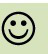	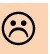
Wang et al. (2017)	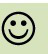	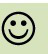	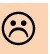	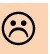	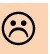	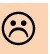
Wang et al. (2021)	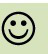	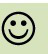	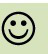	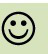	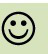	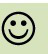
Wang et al. (2022)	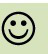	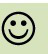	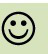	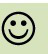	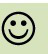	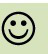
Wu et al. (2018)	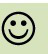	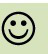	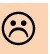	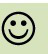	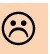	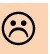
Ye et al. (2017)	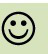	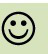	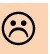	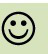	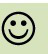	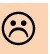
Yu et al. (2016)	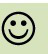	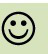	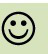	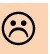	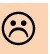	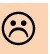
Zhang J. et al. (2025)	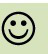	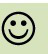	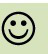	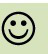	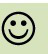	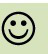
Zhao et al. (2019)	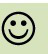	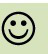	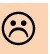	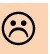	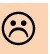	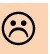
Zhong et al. (2021)	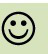	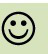	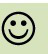	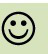	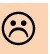	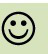

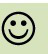
 = low risk; 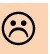 = high risk.

### 3.7 Quality of evidence in the included SRs and MAs

Tables 5–8 present the quality of evidence for the included studies as assessed using the GRADE approach. Across the 19 SRs and MAs, a total of 68 outcome indicators were evaluated. Among these, only 8 outcomes (11.76%) were rated as moderate-quality evidence, 34 (50%) as low-quality, and 26 (38.24%) as critical low-quality. The most frequent reasons for downgrading the quality of evidence were methodological limitations in the included studies, particularly concerning deficiencies in randomization, allocation concealment, and blinding procedure. In addition, 35 outcomes were downgraded due to serious inconsistency, as evidenced by minimal overlap of confidence intervals, statistically significant heterogeneity (low *p-*values), and high I^2^ statistics. Five outcomes were downgraded due to imprecision, such as small sample sizes, wide confidence intervals, or 95% confidence intervals crossing the line of no effect. Furthermore, suspected publication bias contributed to the downgrading of 48 outcomes.

**Table 5:** Effective rate: quality evaluation of included studies by GRADE.

Study	Intervention	Number of RCTs (patients)	Effect (95% CI)	*P*	Risk of bias	Inconsistency	Indirectness	Imprecision	Publication bias	Quality of evidence
Geng et al. (2021)	EA + SRT vs. SRT	14 (1034)	OR: 4.87 (3.47, 6.83)	<0.01	−1^①^	0	0	0	0	Moderate
Huang et al. (2020)	EA + SRT vs. SRT	12 (968)	OR: 5.40 (3.78, 7.72)	<0.01	−1^①^	0	0	0	0	Moderate
Li et al. (2018)	Acupuncture vs. SRT	29 (2190)	RR: 1.33 (1.25, 1.43)	<0.01	−1^①^	−1^②^	0	0	−1^④^	Critically low
Li and Deng (2019)	Acupuncture+SRT vs. SRT	14 (1075)	RR: 1.26 (1.19, 1.34)	<0.01	−1^①^	0	0	0	0	Moderate
Li et al. (2021)	EA + SRT vs. SRT	3 (179)	RR: 1.53 (1.14, 2.06)	<0.01	−1^①^	−1^②^	0	0	−1^⑤^	Critically low
Li et al. (2021)	Acupuncture+SRT vs. SRT	27 (2267)	RR: 1.21 (1.16, 1.25)	<0.01	−1^①^	0	0	0	−1^④^	Low
Li et al. (2024)	Acupuncture (+SRT) vs. SRT	9 (612)	OR: 3.77 (2.23, 6.36)	<0.01	−1^①^	0	0	0	0	Moderate
Long and Wu (2012)	Acupuncture+SRT vs. SRT	72 (6134)	OR: 5.17 (4.18, 6.38)	<0.01	−1^①^	0	0	0	0	Moderate
Lu et al. (2021)	Acupuncture+SRT vs. SRT	36 (2846)	RR: 1.23 (1.19, 1.27)	<0.01	−1^①^	0	0	0	0	Moderate
Tang et al. (2022)	Nape acupuncture+SRT vs. SRT	9 (837)	OR: 3.94 (2.57, 6.04)	<0.01	−1^①^	0	0	0	−1^⑤^	Low
Wang et al. (2017)	Acupuncture+ medication/SRT vs. medication/SRT	11 (973)	OR: 5.78 (4.03, 8.27)	<0.01	−1^①^	0	0	0	−1^⑤^	Low
Wang et al. (2017)	Acupuncture+SRT vs. SRT	19 (1678)	OR: 3.84 (2.91, 5.05)	<0.01	−1^①^	0	0	0	−1^④^	Low
Wang et al. (2021)	Acupuncture+SRT vs. SRT	12 (844)	OR: 4.39 (2.88, 6.69)	<0.01	−1^①^	0	0	0	−1^④^	Low
Wang et al. (2022)	Acupuncture (+SRT) vs. SRT	7 (637)	RR: 1.13 (1.02, 1.25)	<0.01	−1^①^	−1^②^	0	0	−1^⑤^	Critically low
Wu et al. (2018)	Acupressure+SRT vs. SRT	12 (1070)	RR: 1.27 (1.16, 1.39)	<0.01	−1^①^	−1^②^	0	0	−1^④^	Critically low
Ye et al. (2017)	Acupuncture+SRT + CT vs. SRT + CT	62 (4809)	RR: 1.17 (1.13, 1.21)	<0.01	−1^①^	−1^②^	0	0	0	Low
Yu et al. (2016)	Acupuncture+SRT vs. SRT	9 (1017)	OR: 0.36 (0.25, 0.50)	<0.01	−1^①^	0	0	0	−1^⑤^	Low
Zhao et al. (2019)	Acupuncture+SRT + CT vs. SRT + CT	28 (2557)	OR: 4.11 (3.29, 5.13)	<0.01	−1^①^	0	0	0	−1^④^	Low

SRT, swallowing rehabilitation therapy; CT, conventional therapy; EA, electroacupuncture; OR, odds ratio; RR, relative risk; CI, confidence interval; −1, downgrade; 0, not downgrade. ^①^ The design of the included studies have a bias in randomization, allocation concealment, and blinding; ^②^ The confidence interval overlaps less, the *P*-value of heterogeneity test is very small, and I^2^ is larger; ^③^ The sample size is small, or the confidence interval is wide, or 95% CI crossed the invalid line; ^④^ Funnel graph asymmetry; ^⑤^ Fewer studies are included and there may exist a larger publication bias.

**Table 6:** Swallowing function: quality evaluation of included studies by GRADE.

Study	Intervention	Number of RCTs (patients)	Effect (95% CI)	*P*	Risk of bias	Inconsistency	Indirectness	Imprecision	Publication bias	Quality of evidence
Water Swallowing Test (WST)										
Geng et al. (2021)	EA + SRT vs. SRT	3 (196)	MD: −0.54 (−0.88, −0.20)	<0.01	−1^①^	0	0	0	−1^⑤^	Low
Huang et al. (2020)	EA + SRT vs. SRT	3 (196)	MD: −0.78 (−1.07, −0.50)	<0.01	−1^①^	−1^②^	0	0	−1^⑤^	Critically low
Jiang et al. (2022)	Acupuncture vs. SRT	5 (292)	MD: −0.46 (−0.70, −0.22)	<0.01	−1^①^	0	0	0	−1^⑤^	Low
Jiang et al. (2022)	Acupuncture+SRT vs. SRT	16 (1220)	MD: −0.74 (−0.96, −0.52)	<0.01	−1^①^	−1^②^	0	0	0	Low
Li et al. (2021)	Acupuncture+SRT vs. SRT	11 (912)	WMD: −0.69 (−0.78, −0.60)	<0.01	−1^①^	0	0	0	0	Moderate
Lu et al. (2021)	Acupuncture+SRT vs. SRT	8 (929)	MD: −0.75 (−1.11, −0.41)	<0.01	−1^①^	−1^②^	0	0	−1^⑤^	Critically low
Wang et al. (2021)	Acupuncture+SRT vs. SRT	3 (162)	MD: −0.82 (−1.25, −0.39)	<0.01	−1^①^	0	0	0	−1^⑤^	Low
Wu et al. (2018)	Acupressure+SRT vs. SRT	7 (599)	MD: −0.72 (−0.94, −0.50)	<0.01	−1^①^	−1^②^	0	0	−1^⑤^	Critically low
Ye et al. (2017)	Acupuncture+SRT + CT vs. SRT + CT	15 (1264)	MD: −0.79 (−1.11, −0.47)	<0.01	−1^①^	−1^②^	0	0	0	Low
Yu et al. (2016)	Acupuncture+SRT vs. SRT	3 (352)	MD: −0.67 (−0.79, −0.55)	<0.01	−1^①^	−1^②^	0	0	−1^⑤^	Critically low
Zhang B. et al. (2025), Zhang J. et al. (2025), and Zhang D. et al. (2025)	Acupuncture+SRT vs. SRT	10 (774)	MD: −0.72 (−0.96, −0.47)	<0.01	−1^①^	−1^②^	0	0	0	Low
Zhong et al. (2021)	Acupuncture+SRT vs. SRT	11 (2122)	MD: −1.21 (−1.85, −0.57)	<0.01	−1^①^	−1^②^	0	0	0	Low
Videofluoroscopic Swallowing Study (VFSS)										
Geng et al. (2021)	EA + SRT vs. SRT	2 (150)	MD: 1.92 (1.51, 2.33)	<0.01	−1^①^	0	0	0	−1^⑤^	Low
Huang et al. (2020)	EA + SRT vs. SRT	2 (150)	MD: 1.47 (1.11, 1.84)	<0.01	−1^①^	0	0	0	−1^⑤^	Low
Jiang et al. (2022)	Acupuncture+SRT vs. SRT	9 (690)	MD: 1.35 (1.00, 1.71)	<0.01	−1^①^	−1^②^	0	0	0	Low
Li et al. (2024)	Acupuncture (+SRT) vs. SRT	4 (306)	MD: 1.32 (0.08, 2.55)	0.04	−1^①^	−1^②^	0	0	−1^⑤^	Critically low
Lu et al. (2021)	Acupuncture+SRT vs. SRT	5 (354)	MD: 2.53 (1.89, 3.17)	<0.01	−1^①^	−1^②^	0	0	−1^⑤^	Critically low
Tang et al. (2022)	Nape acupuncture+SRT vs. SRT	4 (332)	WMD: 1.33 (1.09, 1.58)	<0.01	−1^①^	−1^②^	0	0	−1^⑤^	Critically low
Zhang J. et al. (2025)	Acupuncture+SRT vs. SRT	9 (697)	MD: 1.49 (0.89, 2.09)	<0.01	−1^①^	−1^②^	0	0	0	Low
Zhong et al. (2021)	Acupuncture+SRT vs. SRT	8 (1228)	MD: 2.26 (1.77, 2.74)	<0.01	−1^①^	−1^②^	0	0	0	Low
Fujishima Dysphagia Scale (FDS)										
Geng et al. (2021)	EA + SRT vs. SRT	2 (228)	MD: 2.09 (0.65, 3.53)	<0.01	−1^①^	−1^②^	0	0	−1^⑤^	Critically low
Huang et al. (2020)	EA + SRT vs. SRT	2 (228)	MD: 1.94 (1.67, 2.22)	<0.01	−1^①^	−1^②^	0	0	−1^⑤^	Critically low
Jiang et al. (2022)	Acupuncture+SRT vs. SRT	4 (280)	MD: 1.31 (0.82, 1.80)	<0.01	−1^①^	−1^②^	0	0	−1^⑤^	Critically low
Lu et al. (2021)	Acupuncture+SRT vs. SRT	3 (275)	SMD: 1.92 (1.30, 2.54)	<0.01	−1^①^	−1^②^	0	0	−1^⑤^	Critically low
Wu et al. (2018)	Acupressure+SRT vs. SRT	1 (64)	MD: 1.25 (0.97, 1.53)	0.01	−1^①^	0	0	0	−1^⑤^	Low
Ye et al. (2017)	Acupuncture+SRT + CT vs. SRT + CT	2 (110)	MD: 1.18 (−0.01, 2.36)	0.05	−1^①^	−1^②^	0	−1^③^	−1^⑤^	Critically low
Zhong et al. (2021)	Acupuncture+SRT vs. SRT	12 (2216)	MD: 1.68 (1.16, 2.20)	<0.01	−1^①^	−1^②^	0	0	0	Low
Standard swallowing assessment (SSA)										
Jiang et al. (2022)	Acupuncture vs. SRT	3 (197)	MD: −3.73 (−6.05, −1.41)	<0.01	−1^①^	−1^②^	0	0	−1^⑤^	Critically low
Jiang et al. (2022)	Acupuncture+SRT vs. SRT	12 (1017)	MD: −3.66 (−4.66, −2.66)	<0.01	−1^①^	−1^②^	0	0	0	Low
Li et al. (2021)	Acupuncture+SRT vs. SRT	6 (584)	WMD: −3.41 (−3.98, −2.84)	<0.01	−1^①^	0	0	0	−1^⑤^	Low
Lu et al. (2021)	Acupuncture+SRT vs. SRT	8 (680)	MD: −4.63 (−5.68, −3.59)	<0.01	−1^①^	0	0	0	−1^⑤^	Low
Tang et al. (2022)	Nape acupuncture+SRT vs. SRT	9 (797)	WMD: −2.57 (−3.51, −1.62)	<0.01	−1^①^	−1^②^	0	0	−1^⑤^	Critically low
Ye et al. (2017)	Acupuncture+SRT + CT vs. SRT + CT	11 (891)	MD: −3.70 (−4.93, −2.48)	<0.01	−1^①^	−1^②^	0	0	−1^⑤^	Critically low
Zhang J. et al. (2025)	Acupuncture+SRT vs. SRT	12 (1096)	MD: −3.64 (−4.72, −2.56)	<0.01	−1^①^	−1^②^	0	0	0	Low
Zhong et al. (2021)	Acupuncture+SRT vs. SRT	13 (2408)	MD: −3.78 (−4.64, −2.91)	<0.01	−1^①^	−1^②^	0	0	−1^④^	Critically low
The dysphagia outcome severity score (DOSS)										
Jiang et al. (2022)	Acupuncture+SRT vs. SRT	2 (200)	MD: 1.24 (−0.45, 2.94)	0.15	−1^①^	−1^②^	0	−1^③^	−1^⑤^	Critically low
Penetration aspiration scale (PAS)										
Li et al. (2024)	Acupuncture (+SRT) vs. SRT	16 (1329)	MD: −1.02 (−1.27, −0.78)	<0.01	−1^①^	0	0	0	0	Moderate
Hyoid bone displacement										
Li et al. (2024)	Acupuncture (+SRT) vs. SRT	6 (624)	MD: 2.02 (0.86, 3.18)	<0.01	−1^①^	−1^②^	0	0	−1^⑤^	Critically low

SRT, swallowing rehabilitation therapy; CT, conventional therapy; MD, mean difference; SMD, standardized mean difference; WMD, weighted mean difference; CI, confidence interval; −1, downgrade; 0, not downgrade. ^①^ The design of the included studies have a bias in randomization, allocation concealment, and blinding; ^②^ The confidence overlaps less, the *P*-value of heterogeneity test is very small, and I ^2^ is larger; ^③^ The sample size is small, or the confidence interval is wide, or 95% CI crossed the invalid line; ^④^ Funnel graph asymmetry; ^⑤^ Fewer studies are included and there may exist a larger publication bias.

**Table 7:** The occurrence of pulmonary infection, pneumonia, and aspiration: quality evaluation of included studies by GRADE.

Study	Intervention	Number of RCTs (patients)	Effect (95% CI)	*P*	Risk of bias	Inconsistency	Indirectness	Imprecision	Publication bias	Quality of evidence
Aspiration pneumonia										
Huang et al. (2020)	EA + SRT vs. SRT	2 (170)	OR: 0.20, (0.06, 0.61)	<0.01	−1^①^	0	0	0	−1^⑤^	Low
Jiang et al. (2022)	Acupuncture+SRT vs. SRT	4 (379)	RR: 0.42, (0.25, 0.70)	<0.01	−1^①^	0	0	0	−1^⑤^	Low
Pulmonary infection										
Li et al. (2021)	Acupuncture+SRT vs. SRT	3 (283)	RR: 0.70, (0.43, 1.12)	0.45	−1^①^	0	0	−1^③^	−1^⑤^	Critically low
Wu et al. (2018)	Acupressure+SRT vs. SRT	3 (259)	RR: 0.56 (0.30–1.04)	0.96	−1^①^	0	0	−1^③^	−1^⑤^	Critically low
Zhao et al. (2019)	Acupuncture+SRT + CT vs. SRT + CT	3 (343)	OR: 0.64 (0.33, 1.23)	0.64	−1^①^	0	0	−1^③^	−1^⑤^	Critically low
Aspiration										
Jiang et al. (2022)	Acupuncture+SRT vs. SRT	2 (180)	RR: 0.55, (0.34, 0.90)	0.02	−1^①^	0	0	0	−1^⑤^	Low

SRT, swallowing rehabilitation therapy; CT, conventional therapy; OR, odds ratio; RR, relative risk; CI, confidence interval; −1, downgrade; 0, not downgrade. ^①^ The design of the included studies have a bias in randomization, allocation concealment, and blinding; ^②^ The confidence overlaps less, the *P*-value of heterogeneity test is very small, and I ^2^ is larger; ^③^ The sample size is small, or the confidence interval is wide, or 95% CI crossed the invalid line; ^④^ Funnel graph asymmetry; ^⑤^ Fewer studies are included and there may exist a larger publication bias.

**Table 8:** Quality of life and daily living activities: quality evaluation of included studies by GRADE.

Study	Intervention	Number of RCTs (patients)	Effect (95% CI)	*P*	Risk of bias	Inconsistency	Indirectness	Imprecision	Publication bias	Quality of evidence
Swallowing quality of life questionnaire (SWAL-QOL)										
Jiang et al. (2022)	Acupuncture+SRT vs. SRT	9 (794)	MD: 19.04 (14.08, 24.01)	<0.01	−1^①^	−1^②^	0	0	−1^⑤^	Critically low
Zhang J. et al. (2025)	Acupuncture+SRT vs. SRT	8 (793)	MD: 16.56 (9.94, 23.18)	<0.01	−1^①^	−1^②^	0	0	0	Low
Zhao et al. (2019)	Acupuncture+SRT + CT vs. SRT + CT	4 (381)	MD: 15.31 (11.98, 18.64)	<0.01	−1^①^	0	0	0	−1^⑤^	Low
Barthel index (BI)										
Jiang et al. (2022)	Acupuncture+SRT vs. SRT	2 (205)	MD: 15.99 (12.27, 19.72)	<0.01	−1^①^	0	0	0	−1^⑤^	Low
Modified Barthel index (MBI)										
Zhao et al. (2019)	Acupuncture+SRT + CT vs. SRT + CT	2 (207)	MD: 12.36 (9.17, 15.55)	<0.01	−1^①^	0	0	0	−1^⑤^	Low
Activities of Daily Living (ADL)										
Wu et al. (2018)	Acupressure+SRT vs. SRT	1 (48)	MD: 17.70 (10.31, 25.09)	0.01	−1^①^	0	0	0	-1^⑤^	Low

SRT, swallowing rehabilitation therapy; CT, conventional therapy; MD, mean difference; CI, confidence interval; −1, downgrade; 0, not downgrade. ^①^ The design of the included studies have a bias in randomization, allocation concealment, and blinding; ^②^ The confidence overlaps less, the *P*-value of heterogeneity test is very small, and I^2^ is larger; ^③^ The sample size is small, or the confidence interval is wide, or 95% CI crossed the invalid line; ^④^ Funnel graph asymmetry; ^⑤^ Fewer studies are included and there may exist a larger publication bias.

### 3.8 Efficacy and safety of acupuncture for PSD

#### 3.8.1 Effective rate

A total of 16 SRs and MAs (Geng et al., 2021; Huang et al., 2020; Li et al., 2018, 2021, 2024; Li and Deng, 2019; Long and Wu, 2012; Lu et al., 2021; Tang et al., 2022; Wang et al., 2017, 2021, 2022; Wu et al., 2018; Ye et al., 2017; Yu et al., 2016; Zhao et al., 2019) reported on the clinical effective rate (Table 5). Among these, 11 studies (Li and Deng, 2019; Li et al., 2021, 2024; Long and Wu, 2012; Lu et al., 2021; Wang et al., 2017, 2021, 2022; Ye et al., 2017; Yu et al., 2016; Zhao et al., 2019) demonstrated that acupuncture combined with swallowing rehabilitation therapy (SRT) was significantly more effective than SRT alone. Moreover, acupuncture alone also showed a significant advantage over SRT (RR: 1.33, 95% CI: 1.25–1.43) (Li et al., 2018). Three studies (Geng et al., 2021; Huang et al., 2020; Li et al., 2021) indicated that electroacupuncture combined with SRT resulted in superior clinical efficacy compared to SRT alone. One meta-analysis (Tang et al., 2022), incorporating nine RCTs, found that nape acupuncture combined with SRT was significantly more effective than SRT alone (OR: 3.94, 95% CI: 2.57–6.04). Another study (Wu et al., 2018), synthesizing 12 RCTs involving 1,070 patients, reported that acupressure combined with SRT led to better clinical outcomes than SRT alone (RR: 1.27, 95% CI: 1.16–1.39).

#### 3.8.2 Swallowing function

##### 3.8.2.1 WST

Eleven SRs (Geng et al., 2021; Huang et al., 2020; Jiang et al., 2022; Li et al., 2021; Lu et al., 2021; Wang et al., 2021; Wu et al., 2018; Ye et al., 2017; Yu et al., 2016; Zhang J. et al., 2025; Zhong et al., 2021) analyzed the WST as an outcome measure. Two studies (Geng et al., 2021; Huang et al., 2020) demonstrated that electroacupuncture combined with SRT significantly improved WST scores compared to SRT alone (MD: –0.54, 95% CI: −0.88 to −0.20; MD: –0.78, 95% CI: −1.07 to −0.50). A meta-analysis by Jiang et al. (2022), which included five RCTs reporting on WST, found that acupuncture was more effective than SRT in enhancing WST performance (MD: –0.46, 95% CI: −0.70 to −0.22). Furthermore, eight studies (Jiang et al., 2022; Li et al., 2021; Lu et al., 2021; Wang et al., 2021; Ye et al., 2017; Yu et al., 2016; Zhang J. et al., 2025; Zhong et al., 2021) indicated that acupuncture combined with SRT led to greater improvements in WST scores compared to SRT alone. Additionally, Wu et al. (2018) reported that acupressure combined with SRT significantly outperformed SRT alone in improving WST outcomes (MD: –0.72, 95% CI: −0.94 to −0.50), as shown in Table 6.

##### 3.8.2.2 VFSS

Eight studies (Geng et al., 2021; Huang et al., 2020; Jiang et al., 2022; Li et al., 2024; Lu et al., 2021; Tang et al., 2022; Zhang J. et al., 2025; Zhong et al., 2021) evaluated the VFSS as an outcome indicator. The findings indicated that electroacupuncture combined with SRT significantly improved VFSS scores compared to SRT alone (MD: 1.92, 95% CI: 1.51–2.33; MD: 1.47, 95% CI: 1.11–1.84) (Geng et al., 2021; Huang et al., 2020). Five studies (Jiang et al., 2022; Li et al., 2024; Lu et al., 2021; Zhang J. et al., 2025; Zhong et al., 2021) reported that acupuncture plus SRT was more effective than SRT alone in enhancing VFSS outcomes. One study (Tang et al., 2022), based on a pooled analysis of four RCTs involving 332 patients, demonstrated that nape acupuncture combined with SRT was superior to SRT alone (WMD: 1.33, 95% CI: 1.09–1.58), as illustrated in Table 6.

##### 3.8.2.3 FDS

Seven SRs and MAs (Geng et al., 2021; Huang et al., 2020; Jiang et al., 2022; Lu et al., 2021; Wu et al., 2018; Ye et al., 2017; Zhong et al., 2021) evaluated the FDS. One study (Wu et al., 2018) revealed that acupressure combined with SRT led to better outcomes (MD: 1.25, 95% CI: 0.97–1.53). Similarly, two MAs (Geng et al., 2021; Huang et al., 2020) demonstrated that electroacupuncture combined with SRT showed superior improvement in FDS compared to SRT alone (MD: 2.09, 95% CI: 0.65–3.53; MD: 1.94, 95% CI: 1.67–2.22). Three studies (Jiang et al., 2022; Lu et al., 2021; Zhong et al., 2021) reported that acupuncture combined with SRT improved FDS more effectively than SRT alone. However, when compared with SRT plus conventional therapy (CT), acupuncture combined with SRT and CT did not show a statistically significant difference (MD: 1.18, 95% CI: −0.01 to 2.36) (Ye et al., 2017), as displayed in Table 6.

##### 3.8.2.4 SSA

Seven SRs and MAs (Jiang et al., 2022; Li et al., 2021; Lu et al., 2021; Tang et al., 2022; Ye et al., 2017; Zhang J. et al., 2025; Zhong et al., 2021) assessed the SSA indicator. Among them, five studies (Jiang et al., 2022; Li et al., 2021; Lu et al., 2021; Zhang J. et al., 2025; Zhong et al., 2021) found that acupuncture combined with SRT was more effective in improving SSA scores. Furthermore, one MA (Jiang et al., 2022) analyzed three RCTs comparing acupuncture with SRT and indicated that acupuncture led to greater improvements in SSA (MD: –3.73, 95% CI: −6.05 to −1.41). Two additional studies (Tang et al., 2022; Ye et al., 2017) reported that acupuncture combined with SRT (WMD: –2.57, 95% CI: −3.51 to −1.62) and acupuncture combined with SRT and CT (MD: –3.70, 95% CI: −4.93 to −2.48) were more effective in enhancing SSA scores compared to SRT alone and SRT combined with CT, respectively (Table 6).

##### 3.8.2.5 PAS, hyoid bone displacement, and DOSS

Regarding the PAS, a moderate-quality study by Li et al. (2024), involving 1,329 patients, demonstrated that acupuncture (with or without SRT) significantly outperformed SRT in improving PAS scores (MD: –1.02, 95% CI: −1.27 to −0.78). In the same study, a meta-analysis of six RCTs also revealed that acupuncture (with or without SRT) was more effective in enhancing hyoid bone displacement, as shown in Table 6. One study (Jiang et al., 2022) analyzing two RCTs involving 200 patients found that acupuncture combined with SRT did not show a significant advantage over SRT alone in improving DOSS scores (MD: 1.24, 95% CI: −0.45 to 2.94).

#### 3.8.3 Aspiration pneumonia, pulmonary infections, and aspiration

Table 7 summarizes the SRs and MAs reporting on the occurrence of aspiration pneumonia, pulmonary infections, and aspiration. Two studies (Huang et al., 2020; Jiang et al., 2022) evaluated the incidence of aspiration pneumonia and found that electroacupuncture combined with SRT (OR: 0.20, 95% CI: 0.06–0.61) and acupuncture combined with SRT (RR: 0.42, 95% CI: 0.25–0.70) significantly reduced its occurrence. However, acupuncture combined with SRT, acupressure combined with SRT, or acupuncture combined with SRT and CT did not demonstrate a significant advantage in reducing the incidence of pulmonary infections (Li et al., 2021; Wu et al., 2018; Zhao et al., 2019). In terms of aspiration, a meta-analysis (Jiang et al., 2022) including two RCTs with a total of 180 patients indicated that acupuncture combined with SRT significantly reduced aspiration rates (RR: 0.55, 95% CI: 0.34–0.90).

#### 3.8.4 Quality of life and daily living activities

Three SRs and MAs (Jiang et al., 2022; Zhang J. et al., 2025; Zhao et al., 2019) included SWAL-QOL as an outcome measure (Table 8). Two of them Jiang et al. (2022) and Zhang J. et al. (2025) reported that acupuncture combined with SRT was more effective than SRT alone in improving swallowing-related quality of life. The remaining study (Zhao et al., 2019), which analyzed four original RCTs, found that acupuncture combined with SRT and CT was superior in enhancing quality of life (MD: 15.31, 95% CI: 11.98–18.64). Additionally, one study (Jiang et al., 2022) reported improvements in the Barthel Index (BI) through meta-analysis, showing that acupuncture combined with SRT led to better outcomes (MD: 15.99, 95% CI: 12.27–19.72). One study (Zhao et al., 2019) found that acupuncture combined with SRT and CT significantly improved the modified BI compared to SRT and CT alone (MD: 12.36, 95% CI: 9.17–15.55). Another SR (Wu et al., 2018) indicated that, compared to SRT, acupressure combined with SRT was more effective in enhancing ADL scores (MD: 17.70, 95% CI: 10.31–25.09).

### 3.9 Safety outcomes

Eight SRs and MAs (Geng et al., 2021; Huang et al., 2020; Jiang et al., 2022; Li et al., 2024; Tang et al., 2022; Ye et al., 2017; Zhang J. et al., 2025; Zhong et al., 2021) reported adverse events related to acupuncture. These events were generally mild, including pain, discomfort, bleeding, ecchymosis, and subcutaneous hematoma. The remaining eleven SRs did not provide any information regarding safety outcomes.

## 4 Discussion

### 4.1 Summary of the main results

This overview included 19 SRs and MAs published between 2013 and 2025, aiming to evaluate the efficacy of acupuncture in treating PSD. Notably, 10 of these studies were published within the past 5 years, reflecting a growing academic interest in evidence-based support for acupuncture in this field. Using AMSTAR 2, PRISMA-A, ROBIS, and GRADE tools, we assessed the methodological quality, reporting quality, risk of bias, and certainty of evidence from multiple perspectives. None of the included SRs were rated as high quality, and each study exhibited varying degrees of methodological and reporting deficiencies.

Several issues were identified in terms of methodological and reporting quality: (1) Many SRs lacked pre-registration of a study protocol, indicating insufficient prospective planning; (2) Complete and reproducible search strategies were often absent, with few studies conducting manual searches or screening reference lists; (3) Most studies did not provide a detailed list of excluded studies with reasons, reducing the credibility and transparency of the study selection process; (4) Several studies failed to perform standardized assessments of risk of bias and publication bias; (5) The influence of studies with high risk of bias was not adequately discussed or considered in data interpretation; (6) Funding sources and potential conflicts of interest were frequently not reported; and (7) Although these SRs focused on the efficacy of acupuncture for PSD, most failed to report key acupuncture-specific characteristics, such as the Deqi sensation, which is considered critical in clinical practice. These deficiencies likely impaired the overall interpretability of the included SRs. According to the ROBIS analysis, 6 studies (31.58%) were judged to be at low risk of bias, while the remaining 13 were rated as high risk, primarily due to incomplete and non-comprehensive search strategies, use of inappropriate bias assessment tools, insufficient discussion of bias, unstable results, and absence of predefined protocols outlining inclusion criteria, data handling procedures, and analysis methods. In addition, the degree of overlap among primary studies was quantified using the CCA via the GROOVE tool. The overall CCA was 2.86%, indicating a slight overlap among included reviews. This low level of redundancy indicates that the included SRs and MAs were largely independent, thereby minimizing the risk of inflated or biased conclusions resulting from duplicated primary studies.

The results suggested that acupuncture combined with SRT significantly improved clinical effective rates, enhanced swallowing-related functional outcomes (including WST, VFSS, FDS, SSA, PAS, and hyoid bone displacement), improved swallowing-related quality of life, and was not associated with significant adverse events. Regarding the overall quality of the evidence for outcomes, GRADE analysis showed that only 11.76% of them were rated as moderate-quality evidence, while 50% were classified as low quality and 38.24% as critically low quality. These findings underscore the need for caution when recommending acupuncture as an adjunctive treatment for PSD. Methodological limitations were prevalent across the included studies, particularly regarding randomization, allocation concealment, and blinding. Importantly, many of these limitations originated from the design and reporting deficiencies of the primary RCTs. For instance, inadequate reporting of allocation concealment in SRs often reflected incomplete descriptions in the original trials. Moreover, the procedural nature of acupuncture makes blinding inherently difficult to implement, particularly for practitioners. These limitations in the primary studies are often a key factor contributing to the downgrading of evidence certainty in SRs and MAs. Therefore, enhancing the overall quality of future SRs and MAs requires not only standardized methodologies but also the rigorous design and transparent reporting of primary RCTs. Inconsistency was observed by minimal overlap of confidence intervals, statistically significant heterogeneity, and high I^2^ statistics. Imprecision was another common reason for downgrading, typically due to small sample sizes, wide confidence intervals, or confidence intervals crossing the line of no effect. Publication bias was suspected due to funnel plot asymmetry and the small number of included studies.

Although acupuncture is generally considered safe when performed by qualified practitioners, this overview found that only 8 of the 19 included SRs and MAs reported safety outcomes. The reported adverse events (such as mild pain, bleeding, and subcutaneous hematoma) were mostly mild and self-limiting. Nevertheless, more than half of the included reviews lacked any reporting on safety, raising concerns about the completeness and transparency of the existing research evidence. Future RCTs and SRs should incorporate standardized reporting of safety outcomes in accordance with guidelines to better inform clinical decision-making.

### 4.2 Mechanisms of acupuncture for treating PSD

The mechanisms by which acupuncture exerts its effects on PSD are complex and remain to be fully elucidated. Current evidence suggests that acupuncture may alleviate PSD by modulating central nervous structures such as the cortical swallowing centers, subcortical structures, and brainstem swallowing centers, as well as by regulating peripheral nerves and muscle groups involved in swallowing (Ke et al., 2024). Studies have shown that electroacupuncture can enhance the transmission of sensory information related to swallowing to the brainstem, thereby activating the swallowing reflex and facilitating motor cortex excitation (Yao et al., 2023). The primary sensorimotor cortex plays a critical role in the control of swallowing. Electroacupuncture at the Lianquan (CV23) acupoint has been found to increase local blood perfusion and enhance neuronal activity in the primary sensory cortex (Yuan et al., 2024). Further research indicates that acupuncture at CV23 may directly stimulate swallowing-related muscles and the peripheral branches of the glossopharyngeal nerve, influence neurons firing in the nucleus tractus solitarius, and reflexively enhance excitability in the medulla, thus facilitating the recovery of the swallowing reflex arc (Liang et al., 2022). Additionally, electroacupuncture targeting the suprahyoid muscles may enhance sensory input through the glossopharyngeal, trigeminal, and vagus nerves, activate paralyzed pharyngeal muscles, promote pharyngeal muscle contraction, and improve neuromuscular control of swallowing (Jin et al., 2022). In summary, as reported in the included SRs (Huang et al., 2020; Jiang et al., 2022; Li et al., 2024), acupuncture may enhance the excitability of the central nervous system, strengthen nerve reflexes, reconstruct the swallowing reflex arc, and promote the coordination of swallowing-related muscle groups, thereby contributing to the recovery of swallowing function in patients with PSD.

### 4.3 Implications for future studies

Given the current limitations, future research could be improved in several key areas. First, the quality of an SR is fundamentally dependent on the quality of the included primary studies. Therefore, future clinical trials should adhere strictly to the CONSORT guidelines, with particular emphasis on the reporting of adverse events and the proper implementation of randomization, allocation concealment, and blinding procedures. Second, when reporting SRs, researchers should rigorously follow the PRISMA-A checklist to ensure methodological rigor and reporting completeness. When substantial heterogeneity is detected, its potential sources should be thoroughly investigated, and subgroup analyses should be conducted where appropriate to clarify the origins of heterogeneity. In addition, greater emphasis should be placed on risk of bias assessments, with detailed justification and analysis provided to enhance the interpretability and credibility of the findings. Finally, future clinical studies are encouraged to incorporate well-designed sham acupuncture control groups to more accurately assess the specific therapeutic effects of acupuncture. The use of neuroimaging techniques, such as functional magnetic resonance imaging (fMRI) and diffusion tensor imaging, is also recommended to further elucidate the central mechanisms underlying acupuncture’s effects in patients with PSD.

### 4.4 Strength and limitations

To the best of our knowledge, this is the first overview that systematically evaluates SRs and MAs on acupuncture for PSD using multiple standardized assessment tools. This study was conducted based on a pre-registered protocol, which helped minimize the potential risk of bias and enhance the credibility of the findings. Furthermore, we comprehensively assessed and synthesized the methodological quality, reporting quality, risk of bias, and overall certainty of the evidence in existing SRs and MAs on acupuncture for PSD. However, this study also has several limitations. First, our inclusion criteria were limited to SRs and MAs published in English and Chinese, which may have resulted in the omission of relevant studies published in other languages. Second, many of the included SRs and MAs exhibited a high risk of bias, raising concerns about the reliability and robustness of the conclusions regarding acupuncture’s efficacy for PSD. Third, the quality of the original RCTs included in these reviews varied considerably, contributing to the overall low quality of evidence and potentially weakening the strength of support for acupuncture as a treatment for PSD.

## 5 Conclusion

Current evidence indicates that acupuncture, as an important adjunctive therapy for PSD, may help reduce the incidence of aspiration and aspiration pneumonia while improving patients’ swallowing function, quality of life, and ability to perform daily activities. Moreover, acupuncture combined with SRT has shown superior clinical efficacy compared to SRT alone. However, the certainty of evidence for many outcome indicators remains low or critically low. This is primarily due to methodological limitations, incomplete reporting, and potential risks of bias in study selection, data analysis, and result interpretation across existing SRs and MAs. These issues substantially weaken the strength and reliability of the conclusions and limit their applicability in clinical practice. Therefore, rigorously designed and transparently reported RCTs are needed to generate more robust evidence on the efficacy of acupuncture for PSD. Future studies should follow the CONSORT statement, with particular attention to allocation concealment and blinding strategies. The use of sham acupuncture controls or standardized acupuncture protocols is recommended to reduce heterogeneity and enhance reproducibility. Additionally, incorporating neuroimaging techniques such as fMRI may help elucidate the central mechanisms underlying acupuncture’s effects, thereby strengthening the mechanistic rationale and clinical relevance of the findings.

## Data Availability

The original contributions presented in the study are included in the article/Supplementary material, further inquiries can be directed to the corresponding authors.
