# Supplementary material for: The efficacy of acupuncture for post-stroke dysphagia: an overview of systematic reviews and meta-analyses
Source: Front Neurosci. 2025 Aug 28;19:1640471. doi: 10.3389/fnins.2025.1640471 (PMC12423042; doi:10.3389/fnins.2025.1640471)
Supplement: Supplementary file 1 [file Table_1.docx]

**Table S1 Search strategies for eight databases.**

1. **PubMed**

#1 Stroke [Mesh] OR apoplexy [Title/Abstract] OR cerebrovascular accident [Title/Abstract] OR CVA [Title/Abstract] OR brain vascular accident [Title/Abstract] OR brain infarction [Title/Abstract] OR cerebral infarction [Title/Abstract] OR ischemic stroke [Title/Abstract] OR hemorrhagic stroke [Title/Abstract]

#2 Deglutition Disorders [Mesh] OR Dysphagia [Title/Abstract] OR deglutition disorder [Title/Abstract] OR swallowing disorder [Title/Abstract] OR swallowing disorders OR swallowing dysfunction [Title/Abstract] OR Oropharyngeal Dysphagia [Title/Abstract] OR Esophageal Dysphagia [Title/Abstract]

#3 #1 AND #2

#4 Acupuncture [Mesh] OR Acupuncture therapy [Mesh] OR Acupuncture Points [Mesh] OR Moxibustion [Mesh]

#5 Manual acupuncture [Title/Abstract] OR Electroacupuncture [Title/Abstract] OR auricular acupuncture [Title/Abstract] OR scalp acupuncture [Title/Abstract] OR Needle pricking [Title/Abstract] OR Pricking therapy [Title/Abstract] OR warm needling [Title/Abstract] OR Point application [Title/Abstract] OR acupoint application [Title/Abstract] OR Acupotomy [Title/Abstract] OR Trigger point [Title/Abstract] OR De qi [Title/Abstract] OR acupressure [Title/Abstract]

#6 #4 OR #5

#7 #3 AND #6

#8 Systematic Review [Publication Type] OR Meta-Analysis [Publication Type] OR Meta-Analysis as Topic [Mesh] OR Systematic Reviews as Topic [Mesh] OR meta [Title/Abstract] OR meta-analysis [Title/Abstract] OR systematic review [Title/Abstract] OR systematic evaluation [Title/Abstract]

#9 #7 AND #8

1. **Web of Science**

TS= (Stroke OR apoplexy OR cerebrovascular accident OR CVA OR brain vascular accident OR brain infarction OR cerebral infarction OR ischemic stroke OR hemorrhagic stroke) AND TS= (Dysphagia OR deglutition disorder* OR swallowing disorder* OR swallowing dysfunction OR oropharyngeal dysphagia OR esophageal dysphagia) AND TS= (Acupuncture OR Acupuncture therapy OR Acupuncture points OR Moxibustion OR Manual acupuncture OR Electroacupuncture OR auricular acupuncture OR scalp acupuncture OR Needle pricking OR Pricking therapy OR warm needling OR Point application OR acupoint application OR Acupotomy OR Trigger point OR De qi OR acupressure) AND TS= (Systematic Review OR Meta-Analysis OR Meta-Analysis as Topic OR Systematic Reviews as Topic OR Meta OR Meta-analysis OR systematic review OR systematic evaluation)

1. **The Cochrane Library**

#1 Mesh descriptor: [Stroke] explode all trees

#2 “apoplexy”:ti,ab,kw OR “cerebrovascular accident”:ti,ab,kw OR “brain vascular accident”:ti,ab,kw OR “brain vascular accident”:ti,ab,kw OR “brain infarction”:ti,ab,kw OR “cerebral infarction”:ti,ab,kw OR “ischemic stroke”:ti,ab,kw OR “hemorrhagic stroke”:ti,ab,kw

#3 #1 OR #2

#4 Mesh descriptor: [Deglutition Disorders] explode all trees

#5 “Dysphagia”:ti,ab,kw OR “deglutition disorder”:ti,ab,kw OR “swallowing disorder”:ti,ab,kw OR “swallowing disorders”:ti,ab,kw OR “swallowing dysfunction”:ti,ab,kw OR “Oropharyngeal Dysphagia”:ti,ab,kw OR “Esophageal Dysphagia”:ti,ab,kw

#6 #4 OR #5

#7 Mesh descriptor: [Acupuncture] explode all trees;

#8 Mesh descriptor: [Acupuncture Therapy] explode all trees;

#9 Mesh descriptor: [Acupuncture Points] explode all trees;

#10 Mesh descriptor: [Moxibustion] explode all trees;

#11 "Manual acupuncture":ti,ab,kw or "Electroacupuncture":ti,ab,kw or "auricular acupuncture":ti,ab,kw or "scalp acupuncture":ti,ab,kw or "Needle pricking":ti,ab,kw or "Pricking therapy":ti,ab,kw or "warm needling":ti,ab,kw or "Point application":ti,ab,kw or "acupoint application":ti,ab,kw or "Acupotomy":ti,ab,kw or "Trigger point":ti,ab,kw or "De qi":ti,ab,kw or "acupressure":ti,ab,kw

#12 #7 OR #8 OR #9 OR #10 OR #11

#13 Mesh descriptor: [Meta-Analysis as Topic] explode all trees;

#14 Mesh descriptor: [Systematic Reviews as Topic] explode all trees;

#15 "Systematic Review*":ti,ab,kw or "Meta-Analysis":ti,ab,kw or "Meta":ti,ab,kw or "meta-analysis":ti,ab,kw or "Systematic evaluation":ti,ab,kw

#16 #13 OR #14 OR #15

#17 #3 AND #6 AND #12 AND #16

1. **Embase**

#1 'Stroke'/exp OR 'apoplexy':ti,ab,kw OR 'cerebrovascular accident':ti,ab,kw OR ‘brain vascular accident’:ti,ab,kw OR ‘brain vascular accident’:ti,ab,kw OR ‘brain infarction’:ti,ab,kw OR ‘cerebral infarction’:ti,ab,kw OR ‘ischemic stroke’:ti,ab,kw OR ‘hemorrhagic stroke’:ti,ab,kw

#2 'Deglutition disorders'/exp OR ‘Dysphagia’:ti,ab,kw OR ‘deglutition disorder’:ti,ab,kw OR ‘swallowing disorder’:ti,ab,kw OR ‘swallowing disorders’:ti,ab,kw OR ‘swallowing dysfunction’:ti,ab,kw OR ‘Oropharyngeal Dysphagia’:ti,ab,kw OR ‘Esophageal Dysphagia’:ti,ab,kw

#3 #1 AND #2

#4 'Acupuncture'/exp OR 'Acupuncture Therapy'/exp OR 'Acupuncture Points'/exp OR 'Moxibustion'/exp

#5 'Manual acupuncture':ti,ab,kw OR 'Electroacupuncture':ti,ab,kw OR 'auricular acupuncture':ti,ab,kw OR 'scalp acupuncture':ti,ab,kw OR 'Needle pricking':ti,ab,kw OR 'Pricking therapy':ti,ab,kw OR 'warm needling':ti,ab,kw OR 'Point application':ti,ab,kw OR 'acupoint application':ti,ab,kw OR 'Acupotomy':ti,ab,kw OR 'Trigger point':ti,ab,kw OR 'De qi':ti,ab,kw OR 'Acupressure':ti,ab,kw

#6 #4 OR #5

#7 'Meta analysis'/exp OR 'Meta analysis (topic) '/exp OR 'Systematic review'/exp OR 'Systematic review (topic) '/exp

#8 'Systematic Review as Topic':ti,ab,kw OR 'Systematic reviews as topic':ti,ab,kw OR 'Systematic Review*':ti,ab,kw OR 'Meta':ti,ab,kw OR 'Meta-analysis':ti,ab,kw OR 'Systematic evaluation':ti,ab,kw

#9 #7 OR #8

#10 #3 AND #6 AND #9

1. **China National Knowledge Infrastructure (CNKI)**

SU=('卒中后吞咽障碍' +'脑卒中后吞咽障碍'+'卒中后吞咽困难'+'脑卒中后吞咽困难') AND SU=('针刺'+'针灸'+'耳针'+'体针'+'电针'+'温针灸'+'腹针'+'舌针'+'头针'+'穴位'+'经络') AND SU=('荟萃分析'+'系统综述'+'系统评价'+'Meta分析')

1. **VIP**

(U=卒中后吞咽障碍 OR U=脑卒中后吞咽障碍 OR U=卒中后吞咽困难OR U=脑卒中后吞咽困难) AND (U=针刺 OR U=针灸 OR U=耳针 OR U=体针 OR U=电针 OR U=温针灸 OR U=腹针 OR U=舌针OR U=头针 OR U=穴位 OR U=经络) AND (U=荟萃分析 OR U=系统综述 OR U=系统评价 OR U=Meta分析)

1. **Wangfang**

(卒中后吞咽障碍 or 脑卒中后吞咽障碍 or 卒中后吞咽困难 or 脑卒中后吞咽困难) and (针刺 or 针灸or耳针or体针or电针or温针灸or腹针or舌针or头针or 穴位or经络) and (荟萃分析 or 系统综述 or 系统评价 or Meta分析)

1. **Sinomed**

#1 "卒中"[不加权:扩展] OR "脑卒中"[常用字段:智能] OR "中风"[常用字段:智能] OR "脑缺血"[常用字段:智能] OR "脑出血"[常用字段:智能]

#2 "吞咽障碍"[不加权:扩展] OR "吞咽困难"[常用字段:智能]

#3 "针刺疗法"[不加权:扩展] OR "针刺"[不加权:扩展] OR "针灸疗法"[不加权:扩展] OR "针刺"[常用字段:智能] OR "针灸"[常用字段:智能] OR "耳针"[常用字段:智能] OR "电针"[常用字段:智能] OR "体针"[常用字段:智能] OR "温针灸"[常用字段:智能] OR "舌针"[常用字段:智能] OR "头针"[常用字段:智能] OR "腹针"[常用字段:智能] OR "穴位"[常用字段:智能] OR "经脉"[常用字段:智能]

#4 "Meta分析"[不加权:扩展] OR "Meta分析"[常用字段:智能] OR "系统评价"[常用字段:智能] OR "荟萃分析"[常用字段:智能] OR "系统综述"[常用字段:智能]

#5 (#1) AND (#2) AND (#3) AND (#4)
